# Distribution and Antibiotic Resistance Patterns of Pathogenic Bacteria in Patients With Chronic Cutaneous Wounds in China

**DOI:** 10.3389/fmed.2021.609584

**Published:** 2021-03-17

**Authors:** Haonan Guan, Wei Dong, Yechen Lu, Minfei Jiang, Di Zhang, Yakupu Aobuliaximu, Jiaoyun Dong, Yiwen Niu, Yingkai Liu, Bingjie Guan, Jiajun Tang, Shuliang Lu

**Affiliations:** ^1^Department of Burn, School of Medicine, Ruijin Hospital, Shanghai Jiaotong University, Shanghai, China; ^2^Wound Healing Center, School of Medicine, Ruijin Hospital, Shanghai Jiaotong University, Shanghai, China; ^3^Department of General Surgery, School of Medicine, Shanghai General Hospital, Shanghai Jiaotong University, Shanghai, China

**Keywords:** pathogen, bacteria distribution, antibiotic resistance, multi drug resistant, chronic wounds

## Abstract

**Background:** To determine the distribution and antimicrobial susceptibility pattern of pathogenic bacteria in patients with chronic cutaneous wounds on a national scale.

**Methods:** A retrospective study was conducted using the data recorded between January 1, 2018 and January1, 2020 in 195 hospitals across China. After screening the data, 815 patients with chronic wounds were finally analyzed. The data collected included information about the patients' general condition and local cutaneous wound assessments, especially microbial culture and antibiotic susceptibility tests. The analyses were performed using SPSS Version 26.

**Results:** The study included 815 patients (290 [35.6%] females; 63 [50–74] years). The most common causes of chronic cutaneous wounds were diabetes (183, 22.5%), infection (178, 21.8%), and pressure (140, 17.2%). Among these, 521(63.9%) samples tested yielded microbial growth, including 70 (13.4%) polymicrobial infection and 451 (86.6%) monomicrobial infection. The positive rate of microbial culture was highest in wound tissue of ulcers caused by infection (87.6%), followed by pressure (77.1%), diabetes (68.3%), and venous diseases (67.7%). Bates-Jensen wound assessment tool (BWAT) scores >25 and wounds that lasted for more than 3 months had a higher positive rate of microbial culture. BWAT scores >25 and wounds in the rump, perineum, and feet were more likely to exhibit polymicrobial infection. A total of 600 strains were isolated, of which 46.2% (277 strains) were Gram-positive bacteria, 51.3% (308 strains) were Gram-negative bacteria, and 2.5% (15 strains) were fungi. The most common bacterial isolates were *Staphylococcus aureus* (29.2%), *Escherichia coli* (11.5%), *Pseudomonas aeruginosa* (11.0%), *Proteus mirabilis* (8.0%), and *Klebsiella pneumoniae* (5.8%). The susceptibility tests showed that 116 cultured bacteria were Multidrug resistant (MDR) strains. The resistance rates of *S. aureus* were 92.0% (161/175) to penicillin, 58.3% (102/175) to erythromycin, and 50.9% (89/175) to clindamycin. Vancomycin was the most effective antibiotic (0% resistance rate) against all Gram-positive bacteria. Besides, the resistance rates of *E. coli* were 68.1% (47/69) to ampicillin, 68.1% (47/69) to ciprofloxacin, 60.9% (42/69) to levofloxacin. However, all the isolated Gram-negative bacteria showed low resistance rates to tigecycline (3.9%) and amikacin (3.6%).

**Conclusions:** The distribution of bacteria isolated from chronic cutaneous wounds varies with the BWAT scores, causes, duration, and the location of wounds. Multidrug resistance is a serious health issue, and therefore antibiotics used in chronic wounds must be under strict regulation. Our findings may help clinicians in making informed decisions regarding antibiotic therapy.

## Introduction

Cutaneous wound healing is an incredibly complex and regulated process. A chronic cutaneous wound may develop when the wound healing process fails to progress in an orderly and timely manner (1, 2). The wound healing process can be delayed or stalled by a myriad of factors, including diabetes, skin infections, arterial and venous diseases, trauma, burn, pressure, and surgery (3–6). The number of patients developing chronic cutaneous wounds is rapidly increasing due to changing lifestyles and aging problems. Chronic cutaneous wounds present a major social and financial burden on both the individual patients and the entire healthcare system worldwide (1, 7).

Bioburden has been identified as one of the major barriers to wound healing (8). Colonization of the wound site by pathogens contributes substantially to the wound chronicity (9–11). Previous studies have shown that, in addition to primary skin infections, wounds caused by diabetes, pressure, venous diseases, and surgery (surgical site infections, SSIs) are more likely to be colonized by pathogenic bacteria (9, 12). Among them, SSIs represent about 15% of all nosocomial infections, and are extremely difficult to treat due to their resistance to multiple antibiotics (13, 14).

Several lines of evidence regarding the prevalence of pathogenic bacteria in chronic wounds have identified *Staphylococcus aureus, Pseudomonas aeruginosa, Enterococcus faecalis*, and *Proteus mirabilis* as the most prevalent bacteria in chronic wounds (9, 10, 15, 16). However, the distribution of pathogens depends on various factors, such as geographic location, causes of wounds, among others (17). China has a huge population living with chronic wounds that vary in types and causes (4). However, till date, no nationwide study has been done to investigate the distribution and antimicrobial susceptibility of pathogens in patients with chronic cutaneous wounds. To this end, this study aimed to investigate the distribution and antimicrobial susceptibility of pathogenic bacteria in patients with chronic cutaneous wounds in China. Our findings may help clinicians in making informed decisions regarding antibiotic therapy.

## Materials and Methods

### Study Identification and Data Extraction

Retrospective analysis of medical information downloaded from the WoundCareLog database and recorded between January 2018 and January 2020 was performed (18). Specifically, information on patients' general features and local cutaneous wounds was captured. The general features included the patient' name, gender, age, home address, hospital department, first admission time, complications, chief complaint, past medical history, and diagnosis. The information concerning local cutaneous wounds included wound classification, duration, wound location, wound photographs, among others. It should be noted that all wounds were classified according to their causes, such as diabetes, infection, pressure, etc. Among them, we defined “infection” as primary skin infections like erysipelas, impetigo, and scabies. Data on microbial culture and antimicrobial susceptibility tests were also gathered. The medical records in the WoundCareLog database were all uploaded by doctors and nurses in 195 cooperative hospitals across China following unified standards.

### Inclusion and Exclusion Criteria

Patients diagnosed with chronic cutaneous wounds were initially included. Then patients with the following characteristics were excluded: duration of wounds less than 1 month; patients with incomplete information on general features or wounds; patients without records of microbial culture and antimicrobial susceptibility tests. These inclusion and exclusion criteria were applied in stages.

### Swab Collection and Culture

Bacterial cultures and antimicrobial susceptibility tests were performed when patients presented with clinical signs of systemic or local infection, including fever, erythema, local warmth, serous exudate, discoloration of granulation tissue, and foul odor (19, 20). Samples were obtained from cutaneous wounds by trained nurses based on a standardized procedure (21). Before sample collection, wounds were cleansed with sterile normal saline. Excess saline was carefully removed using sterile gauze, the specimens were collected with a sterile swab by swabbing at the middle of the wounds for 5 s under sufficient pressure, and the swab was immediately inserted into a sterile tube and sent to the laboratory within 2 h. Swabs were streaked on MacConkey Agar (MCA), Blood Agar (BA) plates and incubated aerobically at 37°C and 5% CO_2_ for 24 h. Plates without bacterial growth were incubated for another 18–24 h.

### Bacteria Identification

Identification of the purified isolated bacteria was performed using the VITEK MS automated system (bioMérieux, Marcy I'Etoile, France), VITEK 2 COMPACT System (bioMérieux, Marcy I'Etoile, France), or MALDI Biotyper System (Bruker Daltonics GmbH, Bremen, Germany) according to manufacturer's instructions.

### Antimicrobial Susceptibility Test

The drug susceptibility tests were performed using the VITEK2 COMPACT System (bioMérieux, MarcyI'Etoile, France) according to manufacturer's instructions. The results were interpreted based on the guidelines of Clinical and Laboratory Standards Institute (CLSI) (22). Multidrug resistance (MDR) bacteria was defined as bacteria strains that exhibited non-susceptibility to at least one agent in three or more specified categories of antimicrobials (23).

### BWAT Assessment

The status of the wounds was assessed using the Bates-Jensen wound assessment tool (BWAT) (24). Based on the medical data collected from the database, all wounds were rated based on 13 scored items listed in the instructions of BWAT, including wounds size, depth, edges, undermining, necrotic tissue type, necrotic tissue amount, exudate type, exudate amount, skin color surrounding the wound, peripheral tissue edema, peripheral tissue induration, granulation tissue, and epithelialization. The total score was determined by summing up the scores of the 13 items. A higher total score indicated a more severe wound status. All the wounds were independently rated by two researchers. The average value was adopted if the difference between the two scores was less than three; otherwise, the wound was rated by a third researcher who was more experienced.

### Statistical Analysis

Data analyses were performed using SPSS Version 26 (IBM SPSS; Armonk, New York). The age of the patients was expressed as median and interquartile range. Categorical data, such as gender, the result of bacterial culture tests (positive/negative), and the types of bacteria, were presented as frequencies and proportions and were compared using χ2 tests or Fisher exact probability test. A *p*-value of less than 0.05 defined statistical significance.

## Results

### Demographics

**Figure 1:**
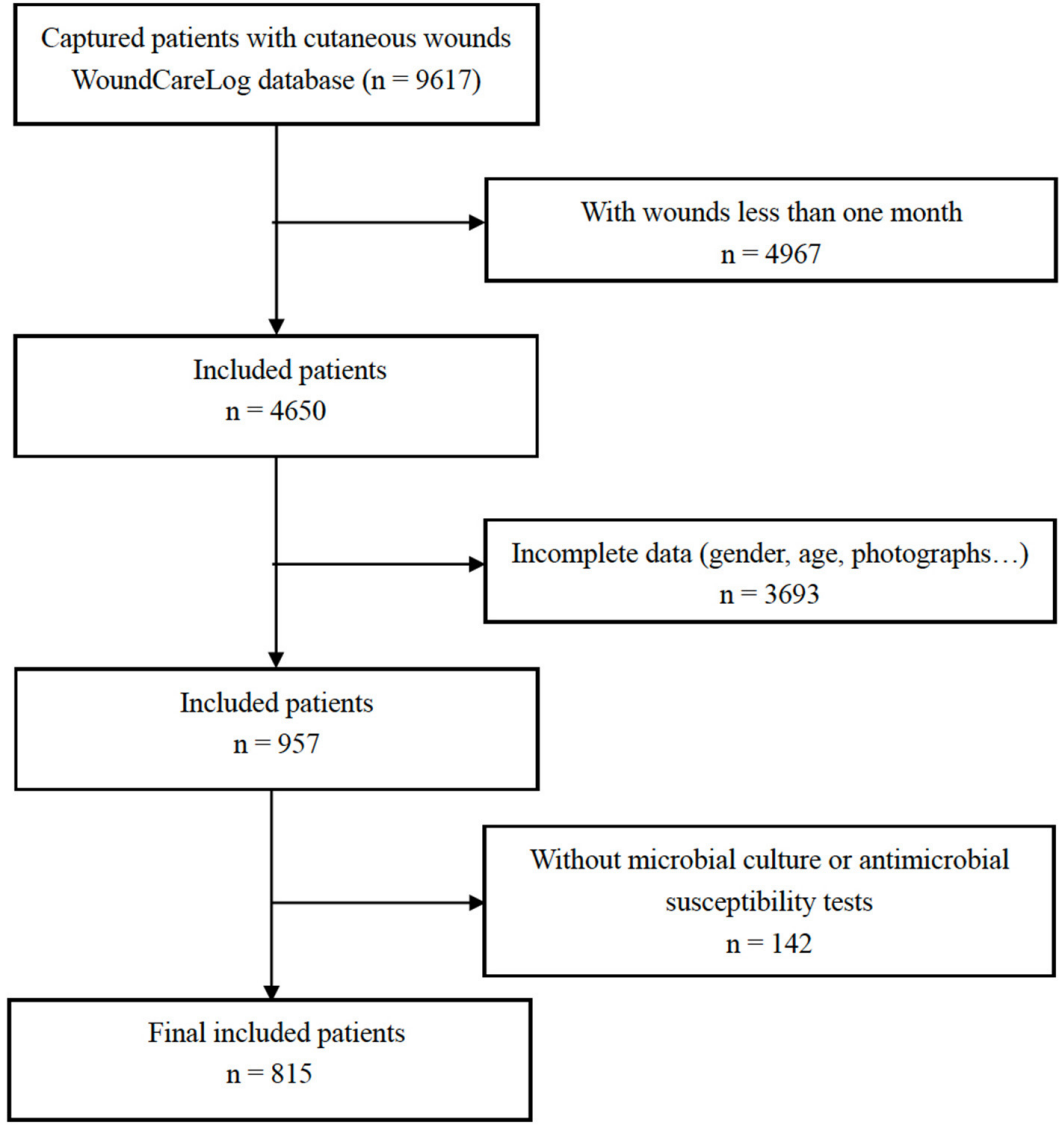
Study selection flowchart.

A total of 38,380 medical records were analyzed, of which 9,617 patients with cutaneous wounds from 195 hospitals across China were identified. Out of which, 8,802 were excluded step by step according to the exclusion criteria, and 815 patients (290 [35.6%] females; median [interquartile range] age, 63 [50–74] years) from 195 hospitals (122 [62.6%] from southern China; 65 [33.3%] from northern China, and 8 [4.1%] from northwestern China) met the inclusion criteria. A flowchart with detailed information was outlined in Figure 1.

In total, 450 (55.2%) patients were over 60 years old. The highest frequency of patients with chronic wounds was found in the age group of 60–80 years (40.9%). Data obtained showed that, most patients came from southern China (75.5%), followed by northern China (23.3%), and only 10 (1.2%) were from northwestern China. The top two most prevalent complications of the analyzed population were diabetes and high blood pressure (HBP), which affected 161 (19.8%) and 121 (14.8%) patients, respectively. The demographic features of the patients are listed in Table 1.

**Table 1:** Participant demographics and clinical variables.

**Variable**	**Subgroup**	**Values, No. (%)**
Age	0–20	20 (2.5)
	21–40	96 (11.8)
	41–60	249 (30.6)
	61–80	333 (40.9)
	>80	117 (14.4)
Gender	Female	290 (35.6)
	male	525 (64.4)
Geographical location	Southern China	615 (75.5)
	Northern China	190 (23.3)
	Northwestern China	10 (1.2)
Complications	Diabetes	161 (19.8)
	HBP	121 (14.8)
	CHD	50 (6.1)
	Nephropathy	13 (1.6)
	PVD	71 (8.7)
	Cerebral infarction	71 (8.7)
Total		815 (100)

*HBP, High Blood Pressure; CHD, Coronary Heart Disease; PVD, Peripheral Vascular Diseases*.

### Wound Information

**Figure 2:**
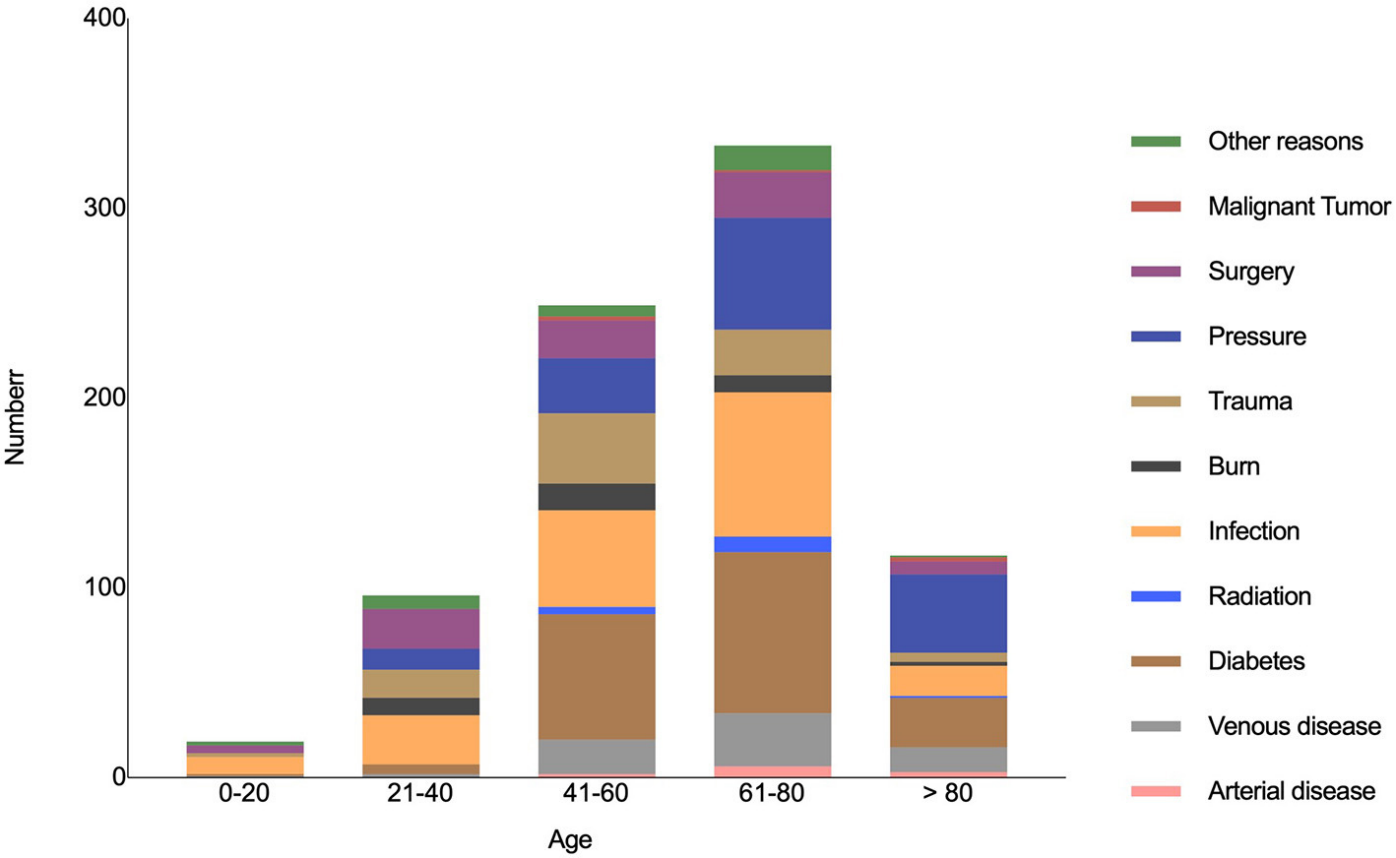
The distribution of different causes in patients of different age groups.

Based on our analysis, chronic cutaneous wounds was caused by diabetes (183, 22.5%), infection (178, 21.8%), pressure (140, 17.2%), trauma (83, 10.2%), surgery (77, 9.4%), venous diseases (62, 7.6%), burn (34, 4.2%), arterial diseases (11, 1.3%), radiation (13, 1.6%), and malignant tumor (5, 0.6%), and other factors (29, 3.6%) including scar ulcers, toxicosis, and autoimmune diseases. The distribution of causes varied significantly in patients in different age groups (χ2 = 49.198, *P* < 0.001) (Figure 2). Other features of chronic cutaneous wounds, such as duration of wounds, BWAT scores, and location of the wounds are listed in Table 2.

**Table 2:** Features of patients' chronic cutaneous wounds.

**Variables**	**Subgroup**	**Values, No. (%)**
Causes	Arterial disease	11 (1.3)
	Venous disease	62 (7.6)
	Diabetes	183 (22.5)
	Radiation	13 (1.6)
	Infection	178 (21.8)
	Burn	34 (4.2)
	Trauma	83 (10.2)
	Pressure	140 (17.2)
	Surgery	77 (9.4)
	Malignant Tumor	5 (0.6)
	Others	29 (3.6)
Duration of wounds(months)	1–3	457 (56.1)
	3–6	143 (17.5)
	6–12	106 (13.0)
	>12	109 (13.4)
BWAT scores	1–25	362 (44.4)
	26–40	411 (50.4)
	>40	42 (5.2)
wounds location	Head and neck	25 (3.1)
	Trunk	156(19.1)
	Rump and perineum	161 (19.7)
	Arm	15 (1.8)
	Hand	19 (2.3)
	Leg	209 (25.6)
	Foot	230 (28.2)
Total		815 (100)

### Microbial Culture

Herein, 63.9% (521 of 815) of samples analyzed yielded microbial growth. The positive rate of microbial culture was significantly higher in patients with cutaneous wounds that lasted for more than 3 months (χ2 = 8.765, *P* = 0.003). Also, the positive rate in BWAT scores > 25 was significantly higher than that of BWAT scores ≤ 25 (χ2 = 13.919, *P* < 0.001). Besides, the positive rate was highest in wound tissue of ulcers caused by infection (87.6%), followed by pressure (77.1%), diabetes (68.3%), and venous diseases (67.7%). Table 3 shows these results. Interestingly, a significant correlation between positive microbial culture and geographical location was observed. Participants from northern China exhibited higher positive rate than those from Southern China (χ2 = 5.099, *P* = 0.024). In total, 451 (86.6%) of the 521 wounds were monomicrobial infections, 70 (13.4%) wounds were polymicrobial infections (≥2 strains were isolated). Similarly, patients with BWAT score > 25 were more likely to have a polymicrobial infection (χ2 = 6.465, *P* = 0.011). Furthermore, we found that the anatomical sites of cutaneous wounds were related to types of infection. Compared to other locations, wounds in the rump, perineum and feet were more likely to have a polymicrobial infection (χ2 =9.897, *P* = 0.002).

**Table 3:** The distribution of common pathogenic bacteria in wounds of different causes.

	**Value**, ***n*** **(%)**											
	**Arterial**	**Venous**	**Diabetes**	**Radiation**	**Infection**	**Burn**	**Trauma**	**Pressure**	**Surgery**	**Malignant**	**Others**	**Total**
	**disease**	**disease**								**tumor**		
Total samples	11	62	183	13	178	34	83	140	77	5	29	815
Positive samples	6 (54.5)	42 (67.7)	125 (68.3)	8 (61.5)	156 (87.6)	18 (52.9)	34 (41.0)	108 (77.1)	18 (23.4)	3 (60.0)	3 (10.3)	521 (63.9)
Total strains	8	47	145	8	173	19	34	139	20	4	3	600
Polymicrobial infection	2 (33.3)	5 (11.9)	16 (12.8)	0 (0)	16 (10.3)	1 (5.6)	0 (0)	27 (25.0)	2 (11.1)	1 (33.3)	0 (0)	70 (11.7)
Monomicrobial infection	4 (66.7)	37 (88.1)	109 (87.2)	8 (100)	140 (89.7)	17 (94.4)	34 (100)	81 (75.0)	16 (88.9)	2 (66.7)	3 (100)	451 (75.2)
MDR	0 (0)	7 (14.9)	31 (21.4)	1 (12.5)	38 (22.0)	2 (10.5)	7 (20.6)	27 (19.4)	2 (10.0)	0 (0)	1 (33.3)	116 (19.3)
Gram-positive bacteria	1 (12.5)	22 (46.8)	73 (50.3)	6 (75.0)	87 (50.3)	13 (68.4)	18 (52.9)	47 (33.8)	7 (35.0)	1 (25.0)	2 (66.7)	277 (46.2)
*S. aureus*	0 (0)	18 (38.3)	51 (35.2)	4 (50.0)	58 (33.5)	7 (36.8)	6 (17.6)	25 (18.0)	4 (20.0)	0 (0)	2 (66.7)	175 (29.2)
MRSA	0 (0)	2 (4.3)	20 (13.8)	1 (12.5)	22 (12.7)	1 (5.3)	3 (8.8)	11 (7.9)	1 (5.0)	0 (0)	1 (33.3)	62 (10.3)
Enterococcus spp.	0 (0)	0 (0)	8 (5.5)	0 (0)	5 (2.9)	1 (5.3)	4 (11.8)	8 (5.8)	1 (5.0)	0 (0)	0 (0)	27 (4.5)
Gram-negative bacteria	7 (87.5)	23 (48.9)	67 (46.2)	2 (25.0)	82 (47.4)	6 (31.6)	15 (44.1)	89 (64.0)	13 (65.0)	3 (75.0)	1 (33.3)	308 (51.3)
*E. coli*	1 (12.5)	4 (8.5)	11 (7.6)	1 (12.5)	18 (10.4)	0 (0)	0 (0)	29 (20.9)	5 (25.0)	0 (0)	0 (0)	69 (11.5)

**Figure 3:**
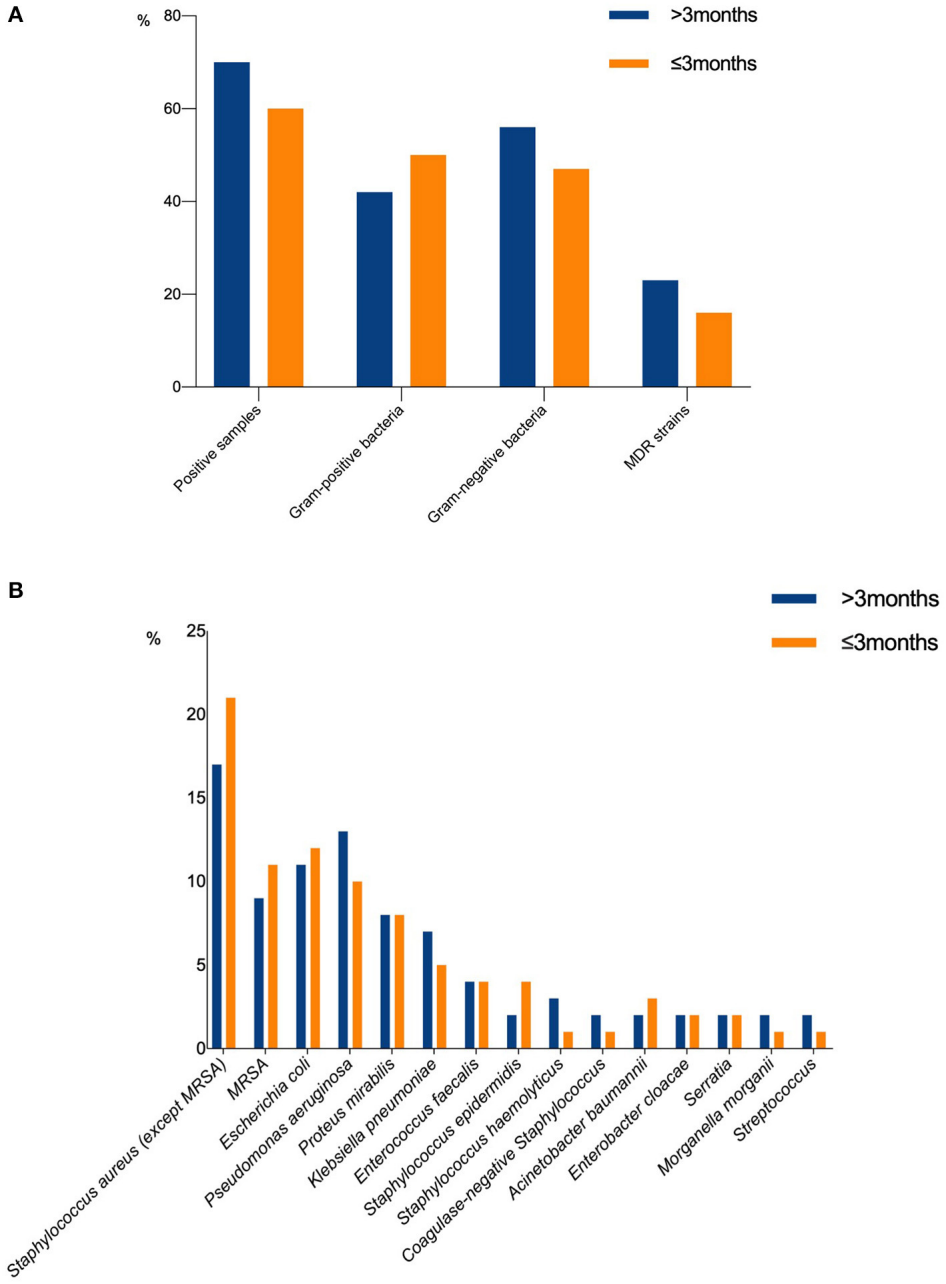
**(A, B)** The distribution of pathogens in wounds of different duration.

A total of 600 bacterial strains were isolated from the 521 cases, 46.2% (277 strains) of which were Gram-positive bacteria, 51.3% (308 strains) were Gram-negative bacteria, and 2.5% (15 strains) were fungi. Samples from the wounds that lasted for more than 3 months mainly contained Gram-negative bacteria, whereas those from the wounds that lasted for less than 3 months mainly contained Gram-positive bacteria (Figure 3A). Besides, wounds caused by radiation and burn were mainly colonized by Gram-positive bacteria, whereas, wounds caused by arterial diseases, pressure, surgery, and malignant tumor were mainly colonized by Gram-negative bacteria. The distribution of common pathogenic bacteria in chronic wounds arising from different causes are listed in Table 3. However, there was no significant association between the distribution of pathogenic bacteria and the age or gender of patients (*P* = 0.527, 0.283, respectively).

**Figure 4:**
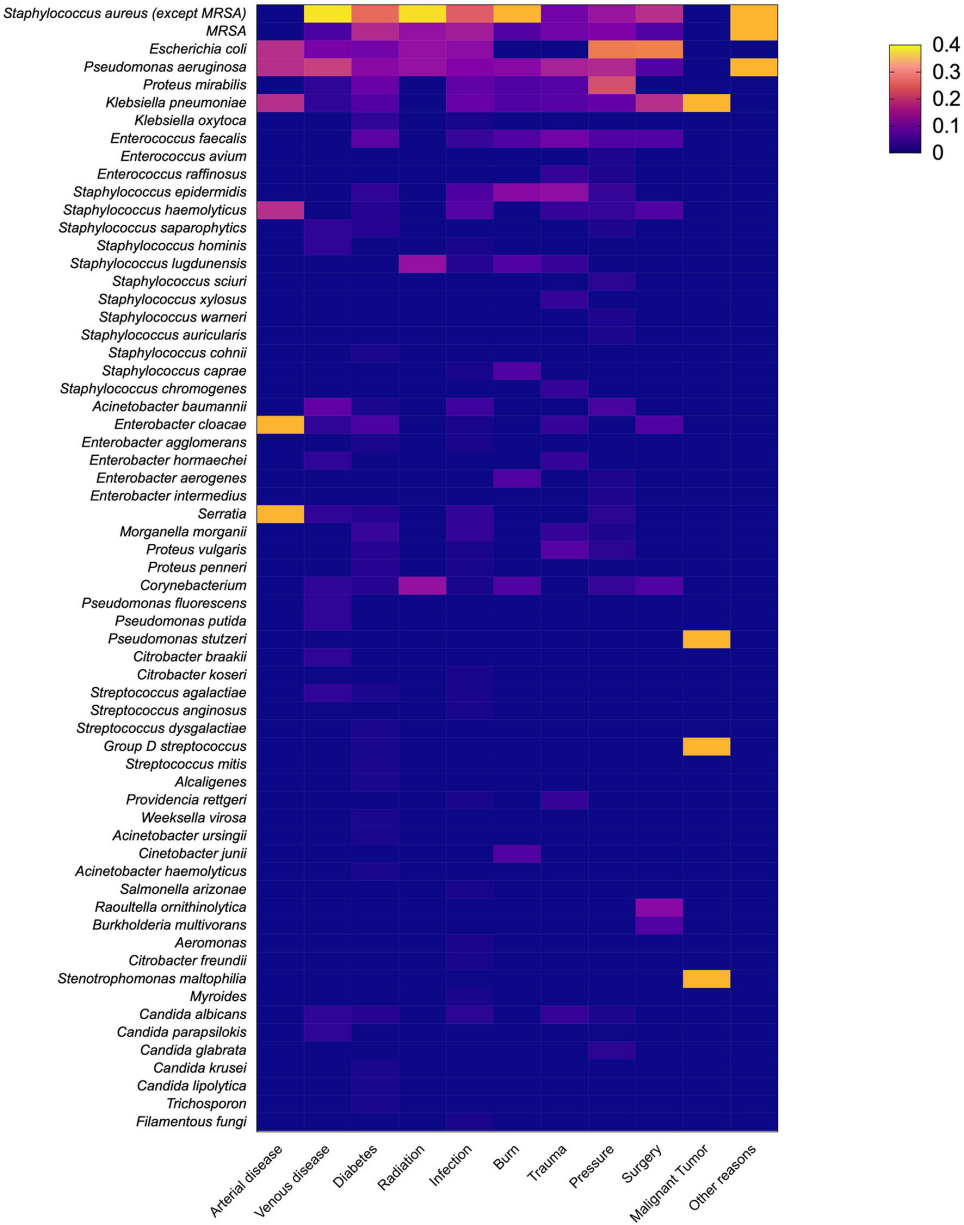
The distribution of pathogens in wounds of different causes. Different colors indicate the percentage of each pathogen in wounds of different etiologies.

The most frequently isolated species were *S. aureus* (29.2%), followed by *E. coli* (11.5%), *P. aeruginosa* (11.0%), *P. mirabilis* (8.0%), and *Klebsiella pneumoniae* (5.8%). In the wounds that formed within 3 months, Gram-positive bacteria played a major role, and 32% of the infections involved *S. aureus*. *E. coli* (12%) was the most common Gram-negative bacteria. However, the wounds that lasted for more than 3 months showed different microbial composition. Gram-negative bacteria accounted for 56.3% of the infections, and *P. aeruginosa* (13%) was the most common Gram-negative bacteria (Figure 3B). The isolated fungi species were as follows: *Candida albicans* (8 strains), *Candida parapsilokis* (1 strain), *Candida glabrata* (2 strains), *Candida krusei* (1 strain), *Candida lipolytica* (1 strain), *Trichosporon* sp. (1 strain), and *Filamentous fungi* (1 strain). However, we did not include fungi in further comparative analysis because of possible bias caused by the small number of isolated fungal strains. The distribution of pathogens in wounds of different causes is presented in Figure 4.

In this study, 22.2% (116 of 521) of the patients developed MDR bacterial colonization, and 116 MDR bacterial strains were cultivated. Among the different causes of chronic cutaneous wounds, MDR bacteria were more likely to be found in wounds caused by infection (22.0%, 38 of 173). The wounds that lasted for more than 3 months had a significantly higher incidence rate of MDR bacterial strains than those that lasted for less than 3 months (χ2 = 4.911, *P* = 0.027). Apart from Methicillin-resistant *Staphylococcus aureus* (MRSA), which was isolated from 62 patients (53.4%), other MDR bacterial species were not common. None of the patients was colonized with vancomycin-resistant *enterococci* (VRE). Among the other 54 patients colonized with MDR bacterial strains, the most common genera were *P. mirabilis* (15, 12.9%), *P. aeruginosa* (13, 11.2%), *Acinetobacter baumannii* (9, 7.8%), *Morganella morganii* (6, 5.2%), *Staphylococcus epidermidis* (5, 4.3%), *E. coli* (4, 3.4%), and *Proteus vulgaris* (2, 1.7%).

### Antimicrobial Susceptibility Test

**Figure 5:**
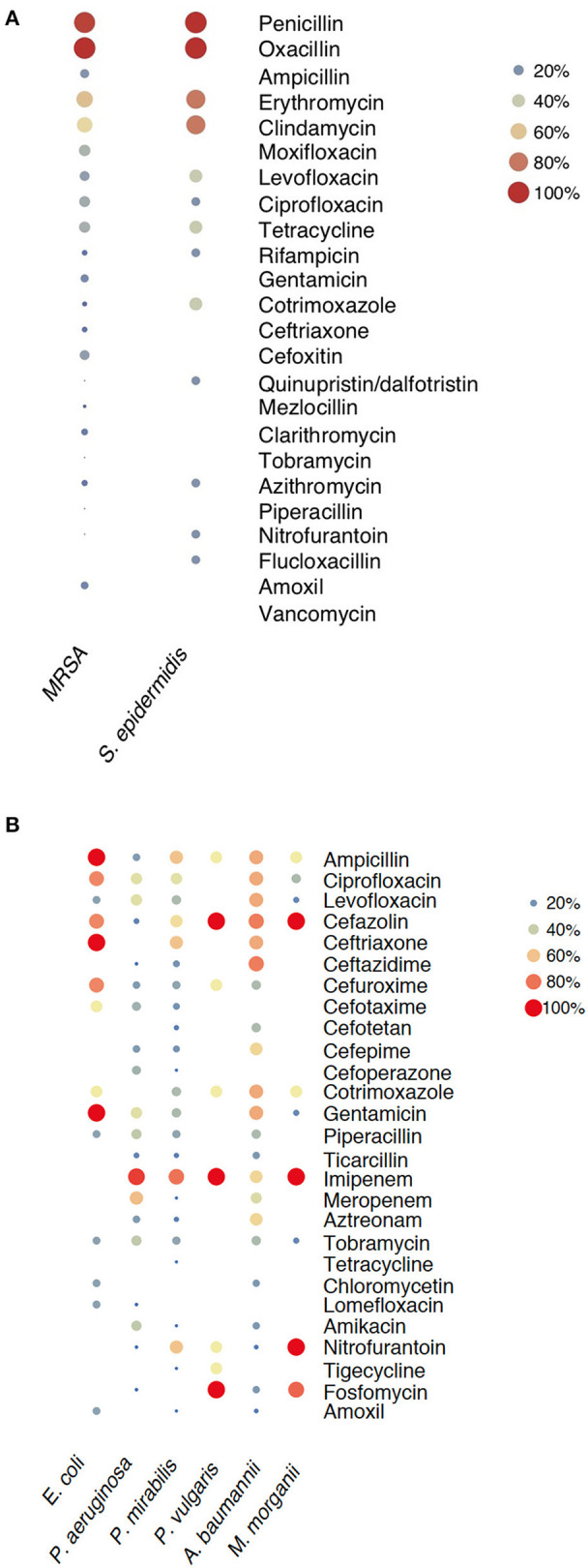
Drug resistance patterns of MDR bacteria. **(A)** Drug resistance patterns of MDR Gram-positive bacteria. **(B)** Drug resistance patterns of MDR Gram-negative bacteria. The color and size of the bubbles indicate the percentage of bacteria that are resistant to certain antibiotics. The blank indicates no resistance.

Details of the antimicrobial resistance pattern of the isolated Gram-positive and Gram-negative bacteria are shown in Tables 4, 5. *S. aureus* showed high resistance rates to penicillin (92.0%), erythromycin (58.3%), and clindamycin (50.9%). The MRSA strains were 100% resistant to oxacillin, followed by penicillin (95%), erythromycin (61.3%), clindamycin (54.8%), and moxifloxacin (32.3%). In contrast, MDR *S. epidermidis* exhibited 100% resistance rates to penicillin, followed by erythromycin (80.0%), clindamycin (80.0%), and levofloxacin (32.3%), but no resistance to moxifloxacin (0%) (Figure 5A). Meanwhile, MDR *E. faecalis* showed high resistance rates to tetracycline (79.2%), quinupristin/dalfotristin (70.8%), and Gentamicin (54.2%). Vancomycin was the most effective antibiotic (0% resistance rate) against all of the Gram-positive bacteria.

**Table 4:** Drug resistance patterns of Gram-positive pathogenic bacteria.

	**Value, No. (%)**				
**Antibiotics**	***S. aureus***	***E. faecalis***	***S. epidermidis***	***S. haemolyticus***	***Streptococcus* spp**.
	**(*n* = 175)**	**(*n* = 24)**	**(*n* = 20)**	**(*n* = 16)**	**(*n* = 8)**
Penicillin	161 (92.0)	6 (25.0)	19 (95.0)	12(75)	1 (12.5)
Oxacillin	61 (34.9)	0 (0)	16 (80.0)	11 (68.8)	0 (0)
Ampicillin	25 (14.3)	5 (20.8)	0 (0)	2 (12.5)	0 (0)
Erythromycin	102 (58.3)	12 (50.0)	15 (75.0)	12(75)	5 (62.5)
Clindamycin	89 (50.9)	7 (29.2)	12 (60.0)	11 (68.8)	8 (100)
Moxifloxacin	17 (9.7)	2 (8.3)	3 (15.0)	8 (50)	0 (0)
Levofloxacin	29 (16.6)	11 (45.8)	8 (40.0)	9 (56.3)	4 (50.0)
Ciprofloxacin	31 (17.7)	11 (45.8)	6 (30.0)	11 (68.8)	0 (0)
Tetracycline	45 (25.7)	19 (79.2)	7 (35.0)	4 (25.0)	1 (12.5)
Rifampicin	10 (5.7)	2 (8.3)	2 (10.0)	5 (31.3)	0 (0)
Gentamicin	31 (17.7)	13 (54.2)	0 (0)	8 (50.0)	0 (0)
Cotrimoxazole	22 (12.6)	2 (8.3)	6 (30.0)	4 (25)	1 (12.5)
Ceftriaxone	7 (4.0)	1 (4.2)	0 (0)	0 (0)	0 (0)
Cefoxitin	15 (8.6)	0 (0)	0 (0)	2 (12.5)	0 (0)
Quinupristin/dalfotristin	1 (0.6)	17 (70.8)	2 (10.0)	1 (6.3)	0 (0)
Amoxil	13 (7.4)	2 (8.3)	0 (0)	2 (12.5)	0 (0)
Vancomycin	0 (0)	0 (0)	0 (0)	0 (0)	0 (0)

**Table 5:** Drug resistance patterns of Gram-negative pathogenic bacteria.

	**Value, No. (%)**							
**Antibiotics**	***E. coli***	***P. aeruginosa***	***P. mirabilis***	***Klebsiella* spp**.	***Enterobacter* spp**.	***A. baumannii***	***Serratia* spp**.	***M. morganii***
	**(*n* = 69)**	**(*n* = 66)**	**(*n* = 48)**	**(*n* = 40)**	**(*n* = 19)**	**(*n* = 15)**	**(*n* = 11)**	**(*n* = 10)**
Ampicillin	47 (68.1)	23 (34.8)	30 (62.5)	18 (45.0)	8 (42.1)	8 (53.3)	4 (36.4)	3 (30)
Ciprofloxacin	47 (68.1)	9 (13.6)	31 (64.6)	16 (40.0)	5 (26.3)	7 (46.7)	0 (0)	3 (30)
Levofloxacin	42 (60.9)	10 (15.2)	17 (35.4)	14 (35.0)	5 (26.3)	7 (46.7)	0 (0)	1 (10)
Cefazolin	30 (43.5)	15 (22.7)	35 (72.9)	18 (45.0)	13 (68.4)	10 (66.7)	8 (72.7)	7 (70)
Ceftriaxone	28 (40.6)	18 (27.3)	29 (60.4)	12 (30.0)	5 (26.3)	6 (40.0)	1 (9.1)	0 (0)
Ceftazidime	12 (17.4)	3 (4.5)	9 (18.8)	6 (15.0)	4 (21.1)	8 (53.3)	0 (0)	0 (0)
Cefuroxime	17 (24.6)	3 (4.5)	14 (29.2)	8 (20.0)	6 (31.6)	4 (26.7)	4 (36.4)	1 (10)
Cefotaxime	7 (10.1)	3 (4.5)	6 (12.5)	4 (10.0)	4 (21.1)	0 (0)	1 (9.1)	0 (0)
Cefepime	11(15.9)	4 (6.1)	11 (22.9)	2 (5.0)	3 (15.8)	5 (33.3)	0 (0)	0 (0)
Cotrimoxazole	28 (40.6)	17 (25.8)	18 (37.5)	24 (60.0)	6 (31.6)	7 (46.7)	2 (18.2)	5 (50)
Gentamicin	18 (26.1)	8 (12.1)	16 (33.3)	12 (30.0)	2 (10.5)	7 (46.7)	1 (9.1)	1 (10)
Piperacillin	18 (26.1)	6 (9.1)	6 (12.5)	4 (10.0)	3 (15.8)	3 (20.0)	2 (18.2)	0 (0)
Imipenem	1 (1.4)	15 (22.7)	9 (18.8)	2 (5.0)	0 (0)	5 (33.3)	0 (0)	8 (80)
Meropenem	0 (0)	9 (13.6)	1 (2.1)	2 (5.0)	1 (5.3)	4 (26.7)	0 (0)	0 (0)
Aztreonam	10 (14.5)	16 (24.2)	8 (16.7)	5 (12.5)	4 (21.1)	9 (60.0)	0 (0)	0 (0)
Tobramycin	10 (14.5)	15 (22.7)	9 (18.8)	5 (12.5)	3 (15.8)	3 (20.0)	0 (0)	1 (10)
Tetracycline	8 (11.6)	7 (10.6)	6 (12.5)	1 (2.5)	1 (5.3)	0 (0)	3 (27.3)	0 (0)
Amikacin	0 (0)	6 (9.1)	3 (6.3)	0 (0)	0 (0)	2 (13.3)	0 (0)	0 (0)
Nitrofurantoin	0 (0)	8 (12.1)	18 (37.5)	11 (27.5)	0 (0)	1 (6.7)	0 (0)	6 (60)
Tigecycline	0 (0)	10 (15.2)	2 (4.2)	0 (0)	0 (0)	0 (0)	0 (0)	0 (0)
Amoxil	5 (7.2)	5 (7.6)	3 (6.3)	0 (0)	0 (0)	2 (13.3)	4 (36.4)	1 (10)

The resistance rates of *E. coli* were 68.1% to ampicillin, 68.1% to ciprofloxacin, and 60.9% to levofloxacin. For *P. aeruginosa*, 34.8% of isolates were resistant to ampicillin, 27.3% to ceftriaxone, and 25.8% were resistant to cotrimoxazole. *P. mirabilis* showed high resistance rates to cefazolin (72.9%), ciprofloxacin (64.6%), and ampicillin (62.5%). However, all the isolated Gram-negative bacteria showed low resistance rates to tigecycline (3.9%) and amikacin (3.6%). In addition, we analyzed antibiotic resistance rates of MDR Gram-negative bacteria, and the results revealed that 49 MDR Gram-negative strains were isolated, which accounted for 15.9% of the Gram-negative bacteria. MDR *E. coli* showed 100% resistance rates to ampicillin, ceftriaxone, and gentamicin whereas MDR *Pseudomonas aeruginosa, Proteus mirabilis, Proteus vulgaris*, and *Morganella morganii* were highly resistant to imipenem (80–100%) (Figure 5B).

## Discussion

Consistent with previous studies (4, 7), this study showed that the most common causes of chronic cutaneous wounds in China were diabetes (22.5%), infection (22.5%), pressure (17.2%), and trauma (10.2%). Comparatively, the primary causes of chronic wounds in western countries are diabetes, venous diseases, pressure, and surgery (12, 25–27). The differences in the etiological characteristics of east and west are, at least in part, a consequence of the differences in economic development, healthcare systems, as well as living and eating habits. Notably, chronic cutaneous wounds have become a major challenge worldwide, and therefore there is an urgent need to develop more effective treatment options.

Microbial infection is the most common challenge to wound healing. A wound is considered infected if the bacteria exceeds a threshold of 10^5^ per gram of wound tissue (28, 29). Chronic wounds are normally colonized by a large collection of pathogenic bacteria that are more likely to form biofilms, and directly contribute to delayed wound healing (9, 30–32). Bacteria that commonly colonize wounds include *S. aureus, P. aeruginosa*, and *E. coli*. These bacterial species usually exert a damaging effect on wound healing (15, 33). Many other species of bacteria in chronic wounds have also been reported, including *Enterobacter cloacae* (17, 34–36), *Citrobacter* sp. (37, 38), *Peptostreptococcus* sp. (39), *Flavobacter* sp. (40), *Serratia* sp. (41, 42), and *Candida* sp. (43–45).

In the present study, the positive rate of microbial culture (63.9%) was much lower than reported by Howell-Jones (82%) and Kassam (91.4%) in western countries (30, 46), and this could be owing to the fact that many patients use antibiotics for self-treatment before seeking medical attention, thus reducing the total positive rate of microbial culture.

Consistent with other studies (9, 32), the results of the present study revealed that chronic cutaneous wounds contained mainly Gram-negative bacteria. The most common species were *S. aureus* (29.2%), *E. coli* (11.5%), P. aeruginosa (11.0%), *P. mirabilis* (8.0%), and *K. pneumoniae* (6.7%). These results were fall in line with the study by Calina et al. (12), which focused on surgical site infections. Meanwhile, we observed a variation in the bacterial species depending on the causes of wounds. Contrary to previous studies (47), wounds caused by diabetes, radiation, trauma, and burn were mainly colonized by Gram-positive bacteria, whereas those caused by vascular diseases, pressure, surgery and malignant tumor were mainly infected with Gram-negative bacteria. The bacteria in chronic wounds mostly form a polymicrobial environment, which provides a suitable environment for genetic exchange between different bacteria and contribute to antibiotic resistance (30). However, the present study illustrated the majority of wound infections were monomicrobial infections (75.2%). In addition, wounds in the rump, perineum, and feet were more likely to form polymicrobial infections than wounds in other body parts.

In the present study, the number of MDR bacterial isolates was 116 from 116 cases, with an occurrence rate of 19.3%. Several studies have reported a 10–59% occurrence rate of MDR bacterial strains in chronic wounds (48–50). We cultivated 62 MRSA strains, accounting for 53.4% of all MDR strains and 35.4% of the *S. aureus* strains. Our findings were consistent with other studies reporting that MRSA take up about 40% of the *S. aureus* strains in SSIs (12, 51). *S. aureus* strains were the most common pathogens in chronic cutaneous wounds and exhibited a high frequency of resistance to antibiotics. Studies have demonstrated that *S. aureus* usually forms biofilms in chronic wounds, thereby causing drug-resistance (52). Similar to findings by Shittu et al. (53) and Shah et al. (54), no strains of *S. aureus*, including those in the MRSA group, showed resistance to vancomycin in our study. However, high levels of resistance to tetracycline (79.2%), quinupristin/dalfotristin (70%), gentamicin (54.2%), and erythromycin (50.0%) were found in *Enterococcus* spp.

In this study, *E. coli* (11.5%) were the most common Gram-negative bacteria in chronic wounds. However, Wong et al. (15) and Gadepalli et al. (47) both reported that *P. aeruginosa* (14.8–16.7%) were the most common Gram-negative bacteria in chronic wounds. Another study indicated that *P. aeruginosa* were more likely to colonize deeper layers of tissue (55). However, all samples were swab cultures instead of deep tissue cultures in the present study, which might have contributed to the differences in the results. We also found that the resistance rates of *E. coli* to ampicillin, ciprofloxacin, and levofloxacin were high (>60%), with no resistant to meropenem, amikacin, nitrofurantoin, and tigecycline.

In addition, we found a lower resistance rate (<5%) of *Klebsiella* spp., *Enterobacter* spp., and *Serratia* spp.to imipenem, but *M. morganii* showed a high resistant rate to imipenem (80%). Gram-negative bacteria have been shown to be highly susceptible to amikacin and meropenem (56, 57). However, the resistivity of *A. baumannii* to amikacin and meropenem were 13.3 and 26.7%, respectively. The lowest resistivity of Gram-negative was found in tigecycline (3.9%) and amikacin (3.6%). Study elsewhere have reported similar findings (58).

An epidemiological study in 2008 indicated that about 78% of patients with chronic cutaneous wounds in China received antimicrobial treatment (7), which is a higher proportion compared to western countries (59, 60). Admittedly, the overuse and misuse of antibiotics is a global problem, which directly contributes to the spreading of antibiotic resistance, especially in China.

Evolving antibiotic resistance has prompted the judicious use of systemic antimicrobials, particularly in treating local infections, such as cutaneous wounds. The use of topical antimicrobials to manage chronic wounds is necessary for controlling wounds infection and even the formation of bacterial biofilms. Topical antibiotic treatments like polymyxin B, silver sulfadiazine are preferred over systemic antibiotic treatments for infected wounds, and antibiotics should be stopped once the wound is clean (61–63). A retrospective study from Hammond et al. (64) indicated that triple antibiotic (polymyxin B, neomycin, bacitracin) ointments can significantly reduce biofilms produced by *S. aureus* and *P. aeruginosa* isolates in burn wounds. It is noteworthy that the use of topical antibiotics can reduce the amount of systemic antibiotics and delay the occurrence of drug resistance. Therefore, antibiotics, especially systemic antibiotics for chronic wounds treatments, must be used under strict control. Furthermore, for chronic wounds, there is a need to perform microbial culture and antibiotics susceptibility tests prior to prescribing antibiotics.

Our study does have some limitations. Firstly, despite being a non-invasive and widely used method, swabs might provide a less truly status of bacterial colonization in the wounds as compared to puncture or tissue biopsy samples if not operated properly. The results of bacteria culture can be affected by colonizing organisms by improper collection, making it difficult to define that this bacterium is infecting wounds or just colonizing them. Secondly, due to the retrospective study design, we were unable to determine the sources of infections (community or hospital acquired) with insufficient information in our database, which was important for epidemiological purposes and impact directly in the antimicrobial resistance rates. Thirdly, as a large-sample research, the data of our study were gathered from 195 hospitals across the country, and the susceptibility profile for some antimicrobials were still reported for those microorganisms showing intrinsic resistance to these agents in a few hospitals. These data were also included in our study which need to be analyzed carefully. Therefore, we suggest these factors should be taken into account in future studies.

However, our findings may help clinicians to establish informed guidelines regarding antibiotic therapy for patients with chronic cutaneous wounds, with the aim to control the infections more effectively and to avoid the overuse and misuse of antibiotics.

## Conclusion

In summary, we analyzed data on the distribution and antimicrobial susceptibility tests of pathogenic bacteria isolated from chronic cutaneous wounds of patients in China. Collectively, we recommend that the antibiotics used in the treatment of chronic wounds should be under strict regulation. Furthermore, there is a need to perform microbial culture and antibiotics susceptibility tests for bacterial isolates from chronic wounds before prescribing antibiotics. Our findings may guide clinicians in making informed decisions regarding antibiotic treatment for patients with chronic wounds.

## Data Availability Statement

The raw data supporting the conclusions of this article will be made available by the authors, without undue reservation.

## Author Contributions

HG, SL, and JT: concept and design. HG, WD, BG, and YL: drafting of the manuscript. HG, MJ, DZ, and YL: statistical analysis. YA, JD, and YN: administrative, technical, or material support. SL: supervision. All authors: acquisition, analysis, or interpretation of data, and critical revision of the manuscript for important intellectual content.

## Conflict of Interest

The authors declare that the research was conducted in the absence of any commercial or financial relationships that could be construed as a potential conflict of interest.
