# Supplementary material for: Distribution and Antibiotic Resistance Patterns of Pathogenic Bacteria in Patients With Chronic Cutaneous Wounds in China
Source: Front Med (Lausanne). 2021 Mar 17;8:609584. doi: 10.3389/fmed.2021.609584 (PMC8010674; doi:10.3389/fmed.2021.609584)
Supplement: Supplementary file 1 [file Table_1.DOCX]

**sTable 1. The distribution of different causes in patients of different age.**

**sTable 2. Drug resistance patterns of MDR Gram-positive bacteria.**

**sTable 3. Drug resistance patterns of MDR Gram-negative bacteria.**

**(We can provide the raw data if necessary.)**

**sTable 1. The distribution of different causes in patients of different age.**

|  | **No. (%)** | | | | | | | | | | |
| --- | --- | --- | --- | --- | --- | --- | --- | --- | --- | --- | --- |
|  | **Arterial disease** | **Venous disease** | **Diabetes** | **Radiation** | **Infection** | **Burn** | **Trauma** | **Pressure** | **Surgery** | **Malignant Tumor** | **Other reasons** |
| 0-20 | 0  (0) | 1  (5.0) | 1  (5.0) | 0  (0) | 9  (45.0) | 0  (0) | 2  (10.0) | 0  (0) | 5  (25.0) | 0  (0) | 2  (10.0) |
| 21-40 | 0  (0) | 2  (2.1) | 5  (5.2) | 0  (0) | 26  (27.1) | 9  (9.4) | 15  (15.6) | 11  (11.5) | 21  (21.9) | 0  (0) | 7  (7.3) |
| 41-60 | 2  (0.8) | 18  (7.2) | 66  (26.5) | 4  (1.6) | 51  (20.5) | 14  (5.6) | 37  (14.9) | 29  (11.6) | 20  (8.0) | 2  (0.8) | 6  (2.4) |
| 61-80 | 6  (1.8) | 28  (8.4) | 85  (25.5) | 8  (2.4) | 76  (22.8) | 9  (2.7) | 24  (7.2) | 59  (17.7) | 24  (7.2) | 1  (0.3) | 13  (3.9) |
| > 80 | 3  (2.6) | 13  (11.1) | 26  (22.2) | 1  (0.9) | 16  (13.7) | 2  (1.7) | 5  (4.3) | 41  (35.0) | 7  (6.0) | 2  (1.7) | 1  (0.9) |

**sTable 2.** **Drug resistance patterns of MDR Gram-positive bacteria.**

|  | **No. (%)** | |
| --- | --- | --- |
| **Antibiotics** | **MRSA**  **(n=62)** | **S. epidermidis**  **(n=5)** |
| Penicillin | 59(95.2) | 5(100) |
| Oxacillin | 62(100) | 5(100) |
| Ampicillin | 13(21.0) | 0(0) |
| Erythromycin | 38(61.3) | 4(80.0) |
| Clindamycin | 34(54.8) | 4(80.0) |
| Moxifloxacin | 20(32.3) | 0(0) |
| Levofloxacin | 15(24.2) | 2(40.0) |
| Ciprofloxacin | 18(29.0) | 1(20.0) |
| Tetracycline | 19(30.6) | 2(40.0) |
| Rifampicin | 6(9.7) | 1(20.0) |
| Gentamicin | 11(17.7) | 0(0) |
| Cotrimoxazole | 5(8.1) | 2(40.0) |
| Ceftriaxone | 6(9.7) | 0(0) |
| Cefoxitin | 15(24.2) | 0(0) |
| Quinupristin/dalfotristin | 1(1.6) | 1(20.0) |
| Mezlocillin | 3(4.8) | 0(0) |
| Clarithromycin | 8(12.9) | 0(0) |
| Tobramycin | 1(1.6) | 0(0) |
| Azithromycin | 7(11.3) | 1(20.0) |
| Piperacillin | 1(1.6) | 0(0) |
| nitrofurantoin | 1(1.6) | 1(20.0) |
| Flucloxacillin | 0(0) | 1(20.0) |
| Amoxil | 10(16.1) | 0(0) |
| Vancomycin | 0 | 0 |

**sTable 3. Drug resistance patterns of MDR Gram-negative bacteria.**

|  | **No. (%)** | | | | | |
| --- | --- | --- | --- | --- | --- | --- |
| Antibiotics | E. coli  (n=4) | Pseudomonas aeruginosa (n=13) | Proteus  mirabilis(n=15) | proteus vulgaris(n=2) | Acinetobacter baumannii (n=9) | Morganella morganii(n=6) |
| Ampicillin | 4(100) | 3(23.1) | 9(60.0) | 1(50.0) | 6(66.7) | 3(50.0) |
| Ciprofloxacin | 3(75.0) | 6(46.2) | 7(46.7) | 0(0) | 6(66.7) | 2(33.3) |
| Levofloxacin | 1(25.0) | 6(46.2) | 5(33.3) | 0(0) | 6(66.7) | 1(16.7) |
| Cefazolin | 3(75.0) | 2(15.4) | 8(53.3) | 2(100) | 7(77.8) | 6(100) |
| Ceftriaxone | 4(100) | 0(0) | 9(60.0) | 0(0) | 6(66.7) | 0(0) |
| Ceftazidime | 0(0) | 1(7.7) | 3(20.0) | 0(0) | 7(77.8) | 0(0) |
| Cefuroxime | 3(75.0) | 3(23.1) | 4(26.7) | 1(50.0) | 3(33.3) | 0(0) |
| Cefotaxime | 2(50.0) | 4(30.8) | 3(20.0) | 0(0) | 0(0) | 0(0) |
| Cefotetan | 0(0) | 0(0) | 2(13.3) | 0(0) | 3(33.3) | 0(0) |
| Cefepime | 0(0) | 3(23.1) | 3(20.0) | 0(0) | 5(55.6) | 0(0) |
| Cefoperazone | 0(0) | 4(30.8) | 1(6.7) | 0(0) | 0(0) | 0(0) |
| Cotrimoxazole | 2(50.0) | 0(0) | 5(33.3) | 1(50.0) | 6(66.7) | 3(50.0) |
| Gentamicin | 4(100) | 6(46.2) | 5(33.3) | 0(0) | 6(66.7) | 1(16.7) |
| Piperacillin | 1(25.0) | 5(38.5) | 4(26.7) | 0(0) | 3(33.3) | 0(0) |
| Ticarcillin | 0(0) | 2(15.4) | 2(13.3) | 0(0) | 2(22.2) | 0(0) |
| Imipenem | 0(0) | 12(92.3) | 12(80.0) | 2(100) | 5(55.6) | 6(100) |
| Meropenem | 0(0) | 8(61.5) | 1(6.7) | 0(0) | 4(44.4) | 0(0) |
| Aztreonam | 0(0) | 3(23.1) | 2(13.3) | 0(0) | 5(55.6) | 0(0) |
| Tobramycin | 1(25.0) | 5(38.5) | 4(26.7) | 0(0) | 3(33.3) | 1(16.7) |
| Tetracycline | 0(0) | 0(0) | 1(6.7) | 0(0) | 0(0) | 0(0) |
| Chloromycetin | 1(25.0) | 0(0) | 0(0) | 0(0) | 2(22.2) | 0(0) |
| Lomefloxacin | 1(25.0) | 1(7.7) | 0(0) | 0(0) | 0(0) | 0(0) |
| Amikacin | 0(0) | 5(38.5) | 1(6.7) | 0(0) | 2(22.2) | 0(0) |
| Nitrofurantoin | 0(0) | 1(7.7) | 9(60.0) | 1(50.0) | 1(11.1) | 6(100) |
| Tigecycline | 0(0) | 0(0) | 1(6.7) | 1(50.0) | 0(0) | 0(0) |
| Fosfomycin | 0(0) | 1(7.7) | 0(0) | 2(100) | 2(22.2) | 5(83.3) |
| Amoxil | 1(25.0) | 0(0) | 1(6.7) | 0(0) | 1(11.1) | 0(0) |
